# A comprehensive survey into the role of microRNAs in ovarian cancer chemoresistance; an updated overview

**DOI:** 10.1186/s13048-022-01012-1

**Published:** 2022-07-07

**Authors:** Ahmad Saburi, Mohammad Saeed Kahrizi, Navid Naghsh, Hasti Etemadi, Ahmet İlhan, Ali Adili, Shadi Ghoreishizadeh, Rozita Tamjidifar, Morteza Akbari, Gülinnaz Ercan

**Affiliations:** 1 Department of Biology, Faculty of Basic Sciences, Gonbad Kavous University, Gonbad Kavous, Iran; 2 Department of Surgery, Alborz University of Medical Sciences, Karaj, Alborz Iran; 3 Department of Pharmacy, Shahid Sadoughi University of Medical Sciences, Yazd, Iran; 4 Department of Biotechnology, Rajiv Gandhi Institute of IT and Biotechnology, Bharati Vidyapeeth University, Pune, India; 5 Department of Medical Biochemistry, Faculty of Medicine, Cukurova University, Adana, Turkey; 6 Senior Adult Oncology Department, Moffitt Cancer Center, University of South Florida, Tampa, Florida USA; 7 Department of Oncology, Tabriz University of Medical Sciences, Tabriz, Iran; 8 Immunology Research Center, Tabriz University of Medical Sciences, Tabriz, Iran; 9 Department of Medical Biochemistry, Faculty of Medicine, Ege University, Izmir, 35100 Turkey; 10 Department of Medical Biotechnology, Faculty of Advanced Medical Sciences, Tabriz University of Medical Sciences, Tabriz, Iran; 11 Department of Stem Cell, Institute of Health Sciences, Ege University, Izmir, 35100 Turkey

**Keywords:** Ovarian Cancer (OC), miRNA, Chemo-resistance, Platinum, Taxane

## Abstract

Ovarian cancer (OC), a frequent malignant tumor that affects women, is one of the leading causes of cancer-related death in this group of individuals. For the treatment of ovarian cancer, systemic chemotherapy with platinum-based drugs or taxanes is the first-line option. However, drug resistance developed over time during chemotherapy medications worsens the situation. Since uncertainty exists for the mechanism of chemotherapy resistance in ovarian cancer, there is a need to investigate and overcome this problem. miRNAs are engaged in various signaling pathways that contribute to the chemotherapeutic resistance of ovarian cancer. In the current study, we have tried to shed light on the mechanisms by which microRNAs contribute to the drug resistance of ovarian cancer and the use of some microRNAs to combat this chemoresistance, leading to the worse outcome of ovarian cancer patients treated with systemic chemotherapeutics.

## Introduction

According to the cancer statistics, there will be 21,410 estimated new ovarian cancer cases and 13,770 estimated ovarian cancer-related deaths in the United States by 2021 [1]. Meanwhile, in Europe, the incidence of new epithelial ovarian cancer cases is nearly 9.5 per 100 000 person-years, and it is the primary cause of mortality among gynecological malignancies [2]. Epithelial ovarian cancer is the most frequent subtype, accounting for nearly 90% of all ovarian cancer cases. Furthermore, it is frequently diagnosed at an advanced stage, leading to a dismal five-year survival rate, even when receiving optimal care [3, 4]. Besides the heterogeneous nature of the disease, the lack of symptoms in the early stages of the disease leads to the diagnosis of nearly two-thirds of patients at a late stage [5]. For a significant proportion of late-stage patients, chemotherapy and cytoreductive surgery combined with biological agents are considered the gold standard of medical care. Patients with advanced OC typically undergo debulking surgery followed by platinum-based chemotherapy, with disease relapse/progression happening in nearly 25% of patients within six months after the preliminary procedure. Also included in this percent estimate denotes the proportion of patients having platinum-resistant tumors (i.e., those resistant to platinum) [5, 6]. The remaining patients will get initial chemotherapy, but most will go through a recurrence within two to three years of treatment [7]. Despite significant advancements in proteomics, genomics, and radiomics, little progress has been made in translating these discoveries into ovarian cancer treatment effective in the clinical setting in recent years. According to recent studies, the survival rate at the most advanced stages is approximately 47.5 percent (or 47.5 percent overall) [8].

For most of the actions listed above, gene expression must be tightly controlled. Epigenetic, genetic, transcriptional, posttranscriptional, and translational mechanisms are all involved in the gene expression regulation at one or more of the prespecified contexts, with a varied spectrum of biological elements being engaged at one or more of the contexts. The regulatory mechanisms are also involved in gene expression regulation at the cellular level.

MicroRNAs are small non-coding RNAs that are 19 to 23 nucleotides in length and regulate gene expression by complementary base-pairing to the three ′ untranslated regions of target mRNA, resulting in direct degradation or transcription inhibition of target mRNA [9–11]. As a result, microRNAs work primarily by inhibiting gene expression, and they regulate roughly thirty percent of the genes in the human genome [12–15]. Many studies have been done on the function of microRNA expression in cancer, particularly in OC [16]. The role of microRNAs (miRNAs) in chemoresistance, metastatic potentials, EMT, and the control of CSCs has been demonstrated in OC research [17, 18]. Although only a few researchers have done a broad miRNA profiling in ovarian cancer intending to discover and validate miRNA expression biological fingerprints that are more than only prognostic and/or predictive, we believe this is a significant step forward [19].

In this review, we intend to evaluate the role of microRNAs in ovarian cancer and how they are related to the resistance of these cancer cells. First, we will take a deep look into the risk factors and therapeutic options, and then we will discuss the mechanisms and pathways by which microRNAs contribute to drug resistance of OC.

## Ovarian cancer progression and genetic alterations

The ovaries are frequently involved in primary peritoneal cancer, similar to epithelial ovarian cancer in terms of symptoms, progression, origin, and treatment [6]. Initially originating on the edges (fimbriae) of the fallopian tubes, epithelial cancer cells are transferred to the ovaries and eventually become cancerous [7]. In its early stages, ovarian cancer symptoms include pelvic/abdominal pain, abdominal heaviness, bloating, back pain, feeling full, vaginal bleeding, or odd vaginal discharges, particularly amid menstrual cycles or after menopause, and uncommon fluctuations in urine or bowel habits. On rare occasions, ovarian cancer develops metastatic characteristics and spreads to other organs, including the colon or bladder [8]. Gene mutations and other genetic variables and carcinogenesis produced by chemicals play an essential part in OC development. Currently, there are two types of ovarian malignancies known: epithelial cancer, which develops inside the surface lining of the ovaries, and non-epithelial cancer, which involves embryonic and structural cells, as well as hormone-producing cells. Mutations in DNA bases in specific genes, generated mainly through genetic causes, can result in OC development. Chemically induced carcinogenesis can also result in the development of ovarian cancer [20, 21]. Inflammation of the cellular membrane has been connected to symptoms containing null gravidity, infertility, and increased ovulatory frequency.

As a result of the greater likelihood of cellular damage and repair, the rate of DNA mutations increases, the most prevalent types of ovarian cancer are epithelial malignancies, further subdivided into four types: endometrioid, serous, mucinous, and clear cell carcinoma [22, 23]. In addition, high-grade serous and low-grade serous ovarian cancer are two types of serous ovarian cancer, with HGSOC (high-grade serous ovarian cancer) accounting for 70 percent to 80 percent of all subtypes of epithelial ovarian cancers and low-grade serous carcinoma accounting for less than 5 percent. Based on fatality, OCCC (ovarian clear cell carcinoma) is the second most prevalent kind of ovarian cancer, responsible for approximately 10–13 percent of all women diagnosed with the disease. Ovarian clear cell carcinoma was initially thought to be connected with endometriosis, the most likely antecedent lesion for the condition [19]; however, this hypothesis must be proven [24]. It begins in the epithelial layer of the ovary, which is the lining of the ovary, just like the majority of ovarian malignancies. Endometrioid carcinoma, associated with endometriosis, is an equally prevalent type of ovarian cancer (10–20 percent of cases) [25]. Mucinous carcinoma, which accounts for merely around 4 percent of all ovarian carcinomas, is most usually detected at an early stage of development [26]. Ovarian tumors that are not epithelial are of minor importance. Germ cell tumors and sex-cord stromal tumors, on the other hand, are more common in younger women and are associated with more acute symptoms than different types of cancer. An ovarian dysgerminoma is the germ cell tumor that occurs most frequently. It has also been explored whether an ovarian tumor with low malignant potential (LMP) or a borderline tumor could be present. It is made up of aberrant ovarian cells and can become malignant, but in most cases, it does not progress to this stage. Different techniques to treat ovarian cancer, such as targeted medicines, ovarian cancer biomarkers, antibodies, and other approaches, have been utilized. Additionally, light has been shed on some theoretical approaches to finding a cure for ovarian cancer, such as combined methods for machine learning and artificial intelligence tools for both diagnosis and prognosis, as well as therapeutic administration.

In addition to inherited mutations that differ in penetrance as well as somatic mutations, hormonal effects associated with an earlier onset of menopause, the association between exposure to environmental hazards as well as associated gynecological factors such as endometriosis, polycystic ovarian syndrome, and pelvic inflammatory disease are all considered risk factors for PCOS. Control and prevention of disease are more complicated [22]. Genomic mutations in the BRCA genes are responsible for around 10–15 percent of all epithelial ovarian cancers; twenty to twenty-five percent of high-grade serous subtypes originate in patients who have germline BRCA mutations [27]. Ovarian and breast cancer, melanoma, pancreatic cancer, and probably serous/serous-like uterine cancer are related to BRCA1 or BRCA2 mutations. There is limited evidence that BRCA1 mutation carriers have a modestly elevated risk of serous endometrial cancer. However, according to the latest research, the clinical implications are still up in the air [15, 28, 29]. Breast and ovarian cancers are linked with a cumulative risk of nearly 72 percent and 44 percent, respectively, for BRCA1 mutation carriers and 69 percent and 17 percent for BRCA2 mutation carriers until 80 years [30]. Over the previous twenty years, it has been assumed that mutations in the BRCA genes were responsible for most hereditary ovarian carcinomas. Sixteen additional genes connected with hereditary ovarian cancer have been revealed as a consequence of next-generation sequencing, resulting in increasing confirmation of correlated uncommon syndromes associated with gynecologic cancers due to the NGS (next-generation sequencing) [31].

## Ovarian cancer therapy

When it comes to advanced ovarian cancer, the first line of treatment is surgery, followed by chemotherapy. Surgery for ovarian cancer debulking determines the stage and adjuvant therapy. Ovarian cancer metastasizes most frequently within the peritoneal cavity. An association between the amount of remaining tumor after debulking surgery and response rates has been demonstrated in various studies [32]. The purpose of surgical debulking is to remove all visible signs of disease from the patient; as a result, recommendations for optimum debulking have been developed. When the largest residual tumor nodule measures less than 1 cm, ideal debulking has occurred, and when the largest residual tumor nodule measures more than 1 cm, suboptimal debulking has occurred [33]. Debulking surgery can be performed between rounds of chemotherapy or neoadjuvant chemotherapy, delivered after initial debulking surgery has been completed. The standard of care for six cycles has been to administer platinum-containing doublet therapy, either intravenously or intraperitoneally (typically with paclitaxel). Most of these individuals will experience a complete clinical response to their treatment. However, recurrence rates are substantial and vary depending on the stage of the disease. The chance of recurrence in patients with stage III or IV disease is 70–75 percent two years after diagnosis, depending on the cancer stage [34]. The start of new symptoms or an increase in CA 125 levels can be indicators of a recurrence of the disease. The term “platinum-sensitive” refers to patients who recur after receiving a platinum dose six months after the initial treatment. These patients often respond to retreatment with platinum doublet therapy. OC patients who relapse after 12 months have an even greater response to platinum-based chemotherapy when treated with it a second time [35]. Early detection of recurrence with growing CA 125 levels is contentious.

In a prospective study, OC Patients with increased CA 125 levels were randomized to receive therapy instantly or at clinical or symptomatic recurrence. The study demonstrated no benefit in survival for individuals who received early treatment, and patients reported a lower quality of life; therefore, treatment based on CA 125 levels is not recommended [36]. After six months of the last dosage of platinum therapy, recurrence is considered platinum-resistant. Paclitaxel, PEGylated liposomal doxorubicin, gemcitabine, topotecan, or experimental treatment are commonly used to treat these patients. These systemic treatments can be used in conjunction with bevacizumab or separately. In the Aurelia Phase III trial, bevacizumab was used with chemotherapy for platinum-resistant ovarian cancer. While the trial found that adding bevacizumab to single-agent chemotherapy resulted in considerably prolonged PFS and ORR, the results also revealed moderately significant drug-related toxicity [37].

Patients who have achieved a complete clinical response after first debulking surgery and consolidation chemotherapy may get maintenance therapy. Maintenance therapy demonstrated minimal improvement and was associated with severe toxicity; therefore, this was previously essentially a physician’s option. A meta-analysis study of eight trials merging chemotherapy regimens found no improvement in overall survival (HR = 1.03) or progression-free survival (HR = 1.06). Furthermore, sustained chemotherapy exposure was linked to cumulative toxicity, which could affect subsequent lines of treatment. The recent discovery of targeted molecular therapies, on the other hand, has led to more maintenance therapy alternatives with lower toxicity and greater therapeutic efficacy [38].

## Drug resistance in ovarian cancer

It is believed that the aggressive nature of advanced ovarian cancer is associated with the progress of resistance to the chemotherapeutic agents to which the patients are exposed during their treatment. In addition to being problematic, recurrence rates are increasingly challenging to treat when sensitivity to chemotherapy utilized in principal treatment begins to wane during subsequent treatment. Some numerous variables and processes determine the susceptibility of cells to medications; as a result, drug resistance cannot be overcome by targeting merely one particular component or pathway. The most important resistance mechanisms in ovarian cancer include increased membrane transporter activity, deregulation of apoptosis, cancer stem cells, autophagy, epigenetics, and the epithelial-mesenchymal transition (EMT). Additional unidentified fundamental causes, on the other hand, could be having a part in the formation of resistant phenotypes [39–44].

DNA damage repair (DDR), intracellular detoxification, Cellular copper transporters, and non-coding RNA-mediated drug resistance are all examples of mechanisms established by ovarian cancer cells to withstand chemotherapy and other treatments. ATP7A and ATP7B are involved in the absorption or efflux of platinum through the copper transporter 1 (CTR1), respectively [45, 46]. It is interesting to note that in terms of their dysregulated expression, the concentration of drugs within ovarian cancer cells decreases, leading to resistance to chemotherapy. Specifically, glutathione S-transferase is an intracellular detoxification enzyme that stimulates the conjugation of glutathione with chemotherapeutic drugs, resulting in the excretion of such conjugated medicines and the elimination of their toxic effects; generally, this procedure contributes to the development of drug resistance in patients with ovarian cancer [47–49]. The cellular DDR mechanism recognizes and repairs damaged DNA to maintain a stable genome, reducing the amount of DNA damage caused by cisplatin exposure [50, 51]. These mechanisms of treatment of OC resistance, as previously indicated, have been thoroughly inspected. The exact process by which non-coding RNA produces drug resistance, on the other hand, remains a mystery [52].

## microRNAs involved in ovarian cancer

**Fig. 1:**
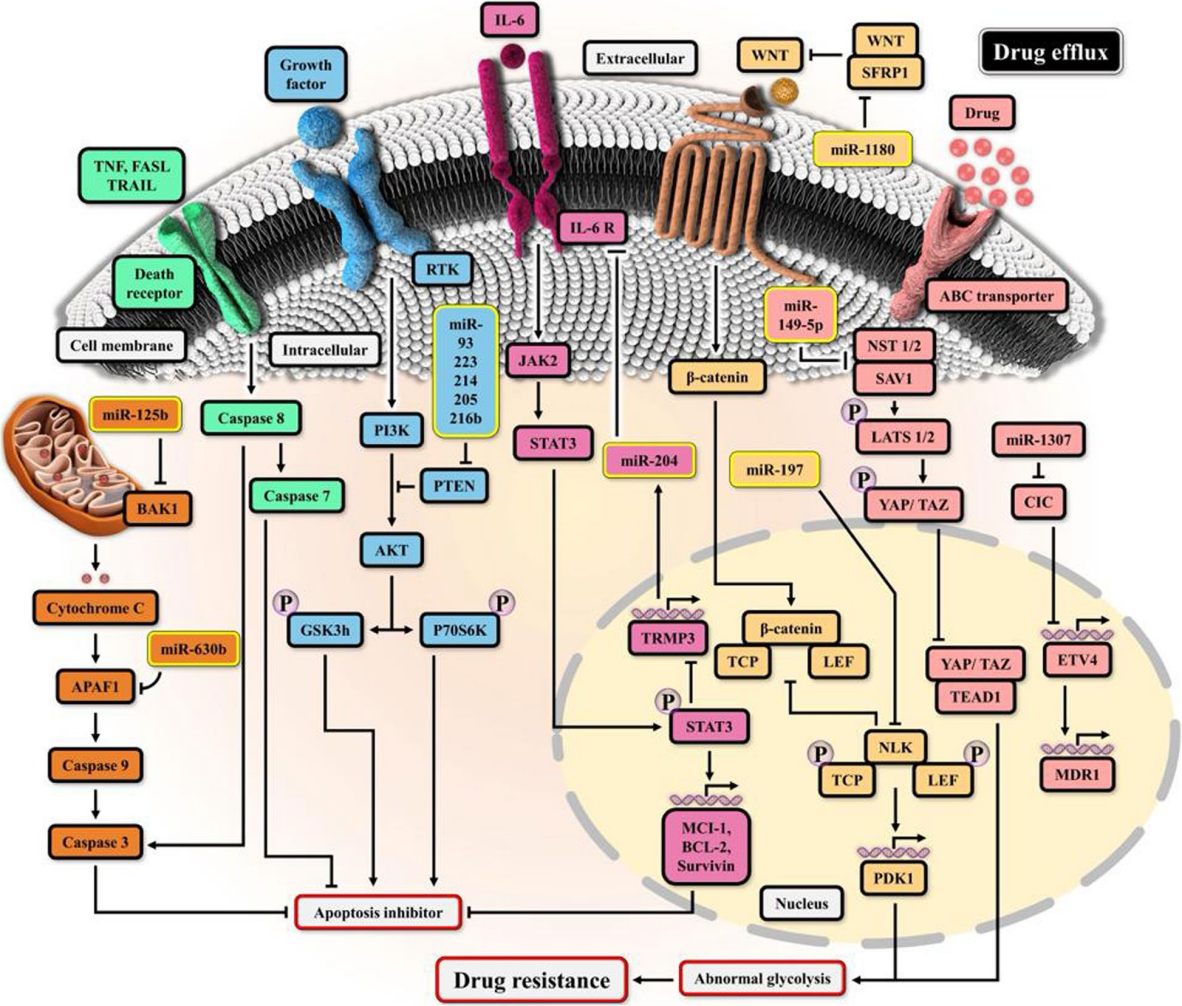
The mechanisms and pathways by which microRNAs contribute to drug resistance in OC which has wholly been summarised in the text

Since the discovery of microRNAs in the worm *C. elegans* in 1993, the field of microRNA research has grown at an exponential rate [53]. A total of 2654 mature microRNAs have been identified in the human genome; miRNAs control the expression of their target genes by interacting with partial complementary three ′ untranslated regions of their target genes [54–57]. Furthermore, extracellular vesicles such as exosomes, which are microscopic vesicles formed of a lipid bilayer and mediate cell-to-cell communication in the local and distant milieu, can encapsulate and distribute miRNAs to their targets [58, 59]. Exosomal transfer of miRNAs and mRNAs can result in oncogenic activities in recipient cells, indicating that miRNAs are essential in cancer progression. These non-coding RNAs are also crucial in preventing cancer [60]. MiRNAs are involved in signaling pathways that contribute to chemotherapeutic resistance. In addition to drug efflux, apoptotic suppression, and aberrant glycolysis, several microRNAs (miRNAs) contribute to drug resistance (Fig. 1).

### microRNAs in OC: expression and function

*In-vitro* and *in-vivo* investigations have revealed that ovarian cancer cells have abnormal miRNA expression. Their participation in essential processes such as cell cycle regulation, apoptosis, cell proliferation, and invasion has been demonstrated in functional investigations. While some microRNAs (miRNAs) have been implicated in the proliferation and invasion of OC, others may play a function in the opposite direction of these processes. miRNA-219-5p has been shown to limit the proliferation, aggression, and migration of EOC by targeting the Twist/Wnt/-catenin signaling pathway, proposing that it may have a role in the diagnosis and therapy of the disease [61]. The traditional Let-7 family of microRNAs has a tumor suppressor role because they target several oncogenic genes. Its expression is downregulated in many tumor cells [62]. Overexpression of Let-7 g leads to a considerable reduction in the development of OC cancer cells. In OVCAR3 and HEY-A8 cells, this effect results in a partial stoppage of the G0/G1 cell cycle and a considerable downregulation of c-Myc and cyclin-D2 expression [63]. Let-7 miRNA family members HMGA2 and LIN28B, along with the RNA-binding protein IGF2BP1, form a self-promoting oncogenic “triangle” capable of effectively counteracting the tumor-suppressing activities of the let-7 miRNA family [64]. In addition to OC, the let-7 antagonistic triangle may be active in a broad spectrum of malignancies, including breast cancer. Targeting let-7 to reduce the potential of this triangle may represent a new path in the diagnosis of early OC. As a result of these findings, it was evident that aberrant miRNA expression may be used to identify new biomarkers for the diagnosis, progression, and monitoring of ovarian cancer [65]. It is also noteworthy that several ovarian cancer-related pathways, including PI3K/Akt, Wnt/-catenin, mTOR, MAPK (Chung et al., 2013), and EGFR, are modulated by microRNAs [66–70]. miR-506 is considered a tumor suppressor miRNA since it directly targets the CDK4 and CDK6 transcription factors. In addition, because FOXM1 is phosphorylated and activated by these CDKs, downregulation of miR-506 results in increased production of CDK4/6 and activation of FOXM1 [71]. As a result of abnormalities in the mitochondria’s oxidative metabolic system, tumor cells produce significantly more reactive oxygen species (ROS) than normal cells.

Furthermore, because tumor cells have a lower degree of oxidative enzyme activity than normal cells, the creation of ROS results in the development of senescence in these cells. In contrast, over-production of FOXM1 results in an increase in the expression of SOD2 and catalase, which lowers ROS levels and delays the onset of senescence [72]. miR-506 has the potential to decrease the production of SNAI2, which is an inhibitor of E-cadherin and an inducer of vimentin in cells. Deregulation of miR-506, as demonstrated by, can result in the induction of the epithelial-mesenchymal transition (EMT) [71]. Ovarian cancer cells had a decreased expression of miR 145. This miRNA suppresses the expression of TRIM2. Bim is degraded in the proteasomes when ERK phosphorylates it, and TRIM2 helps prevent apoptosis by increasing Bim degradation. This is the method through which miR-506 is downregulated, which prevents apoptosis. Besides, TRIM2 up-regulates ERK1/2 and c-FOS, increasing the malignant phenotype of tumor cells [73]. In addition, CFOS and c-JUN upregulate the expression of several genes, one of which is FUT1. Overexpression of FUT1 increases Lewis y levels, promoting the proliferation and invasion of cancer cells [74]. Ovarian cancer is associated with increased expression of miR-551b-3p. This microRNA binds to the STAT3 promoter, recruiting RNA pol II and the transcription factor TWIST1 and boosting STAT3 production [75]. STAT3 increases the expression of many genes related to cancer. Angiogenesis is enhanced by the overexpression of VEGF and HIF-1 caused by STAT3. In addition, STAT3 promotes MMP2 and MMP9 and enhances cancer cell invasiveness. STAT3 also promotes Cyclin D1, c-Myc, and surviving expression to improve cancer cell proliferation and survival [76]. Consistent with the greater incidence of epithelial ovarian cancers (EOCs), most research has evaluated miRNA expression in this form of ovarian cancer. In addition to distinguishing ovarian cancer cells from standard ovarian samples, aberrant expression of microRNAs in ovarian cancer cells is connected with histotype, lymphovascular and organ invasion, and involvement of the ovarian surface [77]. Comparing miRNA expression levels across distinct stages of ovarian cancer has revealed stage-specific patterns. Specifically, Eitan et al. found differential expression of 18 miRNAs, including hsa-miR-449b, between stage I and stage III cases [78].

It is mentioned further down this page what causes miRNA-induced treatment resistance in ovarian cancer. We discuss microRNAs’ signaling pathways and mechanisms contributing to platinum or taxane chemotherapy resistance in OC (Table 1). Ovarian cancer drug resistance is produced by the overexpression of microRNAs or small nuclear RNAs (circRNA) in the cancer cells.

**Table 1:** The ectopic expression of miRNAs and drug resistance pathways

microRNA nomenclature	Drug	Pathway	Mode of drug resistance	Mode of action	Resistance mechanism	**Cell line/ Tissue Sample types**	Ref
**miR-21**	platinum	PTEN	Apoptosis inhibition	Inhibition of mRNA	Downregulation of PTEN leads to drug resistance	SKOV3 and SKOV3/DDP cells	[79]
**miR-93**	Platinum	PTEN/AKT	Apoptosis inhibition	Inhibition of mRNA	Upregulated miR-93 inhibits apoptosis to promote drug resistance via PTEN/AKT pathway	CDDP-sensitive and -resistant ovarian cancer tissue samples and OVCAR3 and SKOV3 cell lines	[80]
**miR-106a**	Platinum	PDCD4/caspase-3, caspase-8	Apoptosis inhibition	Inhibition of mRNA	Upregulated miR-106a inhibits apoptosis to promote drug resistance via the PDCD4/caspase-3 and caspase-8 pathways	OVCAR3 cell line/ CDDP-resistant ovarian cancer OVCAR3/CIS cell line	[81]
**miR-125b**	Platinum	BAK1	Apoptosis inhibition	Inhibition of mRNA	Upregulated miR-125b induces apoptosis inhibition to promote drug resistance via the BAK1 pathway	cisplatin-sensitive ovarian cancer cell line (OV2008) and its resistant variant (C13*)	[82]
**miR-142-5p**	Platinum	MCL-1	Apoptosis inhibition	Inhibition of mRNA	Downregulated miR-142-5p inhibits apoptosis to promote drug resistance via the MCL-1 pathway	OVCAR3, SKOV3 human ovarian cancer cell lines, and HEK293T (293 T) cell line	[83]
**miR-149-5p**	Platinum	MST1, SAV1/YAP, TAZ	Apoptosis inhibition	Inhibition of mRNA	Upregulated miR-149-5p induces apoptosis inhibition, leading to drug resistance via MST1, SAV1/YAP, and TAZ pathways	TOV-21G, A2780, OVCAR-3, Caov-3, ES-2, HO-8910, and SK-OV-3 ovarian cancer cell lines	[84]
**miR-204**	Platinum	IL-6R/STAT3/miR-204/IL-6R	Apoptosis inhibition	Inhibition of mRNA	Downregulated miR-204 in IL-6R/STAT3/miR-204/IL-6R pathway induces apoptosis inhibition to promote drug resistance	The cDDP-resistant EOC cell lines SKOV3, ACRP, OVCAR3, OV2008, and C13*	[85]
**miR-205**	Platinum	PTEN	Apoptosis inhibition	Inhibition of mRNA	Upregulated miR-205 induces apoptosis inhibition to promote drug resistance via the PTEN pathway	110 archived clinical ovarian cancer tissues/six ovarian cancer cell lines (HO-8910, HO-8910PM, SKOV-3, SKOV-3ip, SKOV-3/DDP, and COC1)	[86]
**miR-214**	Platinum	PTEN/AKT	Apoptosis inhibition	Inhibition of mRNA	Upregulated miR-214 inhibits apoptosis to promote drug resistance via PTEN/AKT pathway	Human ovarian cancer cell lines and human immortalized ovarian surface epithelial (HIOSE) cell lines	[17]
**miR-216a**	Platinum	PTEN	Apoptosis inhibition	Inhibition of mRNA	Downregulation of PTEN leads to drug resistance	SKOV3 and OVCA433 cell lines	[87]
**miR-223**	Platinum	PTEN/PI3K/AKT	Apoptosis inhibition	Inhibition of mRNA	Upregulated miR-223 induces apoptosis inhibition to promote drug resistance via PTEN/PI3K/AKT pathway	The human epithelial ovarian cancer cell lines A2780 and SKOV3	[88]
**miR-411**	Platinum	ABCG2	Drug efflux	Inhibition of mRNA	Downregulated miR-411 leads to drug efflux, inducing drug resistance via the ABCG2 pathway	Three-to four-week-old female athymic BALB/c nu/nu mice, SKOV3 and OVCAR3 ovarian cancer cell lines, ten primary ovarian cancer tissues	[89]
**miR-1180**	platinum	SFRP1/Wnt-5a/β-catenin	Glycolysis	Inhibition of mRNA	Upregulated miR-1180 promotes glycolysis to induce drug resistance via SFRP1/Wnt-5a/β-catenin pathway	SKOV3 and COC1 ovarian cancer cells, Surgical specimens, and medical records were collected from 59 ovarian cancer patients	[90]
**miR-27a**	Taxane	HIPK2/MDR1/P-gp	Drug efflux	ceRNA	Downregulation of HIPK2 promotes the transcription of MDR1, thus promoting drug resistance	Human ovarian cancer cell line A2780	[91]
**miR-29b**	Taxane	BAG3/miR-29b/MCL-1	Apoptosis inhibition	Inhibition of mRNA	Downregulated miR-29b in BAG3/miR-29b/MCL-1 pathway induces apoptosis inhibition to promote drug resistance	The ES2 (clear cell carcinoma) and AMOC2 (serous adenocarcinoma) ovarian cancer cell lines	[92]
**miR-106a**	Taxane	Caspase-7; BCL10	Apoptosis inhibition	Inhibition of mRNA	Upregulated miR-106a inhibits apoptosis leading to drug resistance via caspase-7, BCL10 pathway	SKOV3 cell line, Fresh tissue samples from 39 serous ovarian tumors, including six benign serous tumors and 33 malignant serous carcinomas	[93]
**miR-630**	Taxane	APAF-1	Apoptosis inhibition	Inhibition of mRNA	Upregulated miR-630 inhibits apoptosis leading to drug resistance via the APAF-1 pathway	Human epithelial ovarian cancer cell lines SKOV3 and paclitaxel-resistant SKOV3 (SKOV3-TR)	[74]
**miR-1307**	Taxane	CIC/ETV4	Drug efflux	Inhibition of mRNA	Upregulated miR-1307 may lead to drug resistance by promoting MDR1 transcription via CIC/ETV4 pathway	Human ovarian cancer cell lines SKOV3 and SKOV3-TR30	[94]

### ABC transporters and miRNA-induced chemotherapeutic resistance

Numerous miRNAs have been discovered that influence the expression of ABC transporters, which facilitate drug efflux and contribute to chemotherapeutic drug resistance [71, 89, 91, 94]. To be more specific, microRNAs 130a, 1307, and 27a increase the expression of P-glycoprotein in ovarian cancer, resulting in improved treatment resistance (P-gp) [71, 91, 94]. Multidrug resistance-1 (MDR1) encodes a drug transporter called P-gp, known as ABCB1. As a result of its efflux activity, it contributes to drug resistance. Ovarian cancer cells that are resistant to cisplatin are overexpressed with MiR-130a, which indirectly promotes the expression of P-gp. microRNAs miR-27a and miR-1307 are both significantly expressed in paclitaxel-resistant OC cells. By directly downregulating CIC expression, miR-1307 reduces ETV4’s transcriptional repression, whereas ETV4 upregulates MDR1 transcription by binding to the MDR1 promoter region, reversing the mechanical repression of ETV4 [95, 96]. Inhibition of the transcriptional repression of MDR1 by HIPK2 is achieved by targeting miR-27a, which targets the HIPK2 (homeodomain-interacting protein kinase-2) [97]. A decrease in the expression of miR-411, which is mediated by low levels of SLC27A2, results in an increase in ABCG2 expression, which facilitates drug efflux and thus cisplatin resistance in OC patients [89]. ABCC1, ABCB1, and ABCG2 are the ABC transporter family’s most common members, with ABCC1 being the most prevalent. ABCC1 and ABCG2 are ABC transporters that have been documented in fewer investigations involving miRNA and ABC transporters. As a result, additional research into miRNA-induced ABCB1 or ABCG2 expression, which results in ovarian cancer drug resistance, will aid in our knowledge of the mechanism of drug resistance of ABC transporters [52].

### Epithelial-mesenchymal transition and miRNA-mediated chemotherapeutic resistance

Cancers that exhibit malignant activity have been linked to EMT on several occasions. This drug’s effects aid cancer invasion and migration. As discovered by Li et al., miR-181a is overexpressed in paclitaxel-resistant ovarian cancer cells. The overexpression of miR-181a leads to the induction of paclitaxel resistance over the upregulation of N-cadherin and the downregulation of E-cadherin, thereby increasing cell paclitaxel resistance [98]. N-cadherin is a positive regulator of EMT, whereas E-cadherin is a negative regulator of the same process [99]. miRNA-induced EMT may be responsible for the progress of a malignant phenotype in OC, a finding that warrants additional investigation.

### Upregulation of glycolysis and miRNA-mediated chemotherapeutic resistance

Glycolysis is favorable for tumors with a malignant nature because it supplies energy for the metabolism of OC cells, which allows them to become resistant to cisplatin treatment [90, 100]. As a result of stimulating the Wnt signaling pathway and its downstream constituents, including the Wnt5a, beta-catenin, CyclinD, and c-Myc proteins, which can accelerate glycolysis, miR-1180 causes an aberrant elevation of glycolysis. It targets SFRP1 to alleviate its inhibitory influence on Wnt5a, which initiates the Wnt/-catenin signaling pathway, increasing the expression of PDK1 in fibroblasts. PDK1 is a critical enzyme essential for the glycolysis of tumor cells. It is transcribed by the LEF/TCF (lymphoid enhancer factor/T-cell factor) and the beta-catenin gene transcription factors [101]. Cisplatin resistance in OC is a result of this aberrant increase in glycolysis. There has been little research into drug resistance in OC caused by aberrant glycolysis caused by miRNAs; consequently, more research may uncover a novel relationship between drug resistance and miRNAs.

### Inhibition of apoptosis and regulation of chemoresistance by miRs

As the primary cause of OC chemotherapy resistance, the aberrant expression of miRNAs causes apoptosis inhibition, which causes chemotherapeutic resistance. MiR-149-5p, for example, was shown to be significantly expressed in cisplatin-resistant ovarian cancer cells, and deactivation of the Hippo signaling pathway resulted in increased cisplatin resistance by directly suppressing the expression of SAV1 and MST1, according to Xu et al. [84]. The downregulation of SAV1 and MST1 lowers the phosphorylation of TAZ and YAP via inhibiting the phosphorylation of LAST1/2, which decreases the phosphorylation of YAP and TAZ. This increases the nuclear levels of TAZ and YAP, and the overexpression of these proteins inhibits the activity of caspase-3 and caspase-9, resulting in the suppression of apoptosis in the cell. miR-106a is strongly expressed in paclitaxel-resistant and cisplatin-resistant OC cells [81, 93]. Non-invasively reduces the expression of caspase-7 and BCL10 and the level of cleaved caspase-8 and cleaved caspase-3 in the death receptor pathway. As a result, miR-106a inhibits cell death by downregulating the expression of apoptosis-related proteins, making OC cells more resistant to cisplatin and paclitaxel, and other chemotherapy agents. miR-214 targets the PTEN gene in ovarian cancer, causing it to become resistant to cisplatin [17]. When miR-214 is overexpressed in ovarian cancer and PTEN expression is inhibited, the AKT pathway is inhibited, which enhances the phosphorylation of glycogen synthase kinase 3 h (GSK3h) and p70 Ribosomal Protein S6 Kinase (RPSK) in the tumor (p70S6K). As a result, miR-214 enhances apoptosis inhibition, which results in the induction of cisplatin resistance. Furthermore, overexpression of miR-93, miR-216a, or miR-223 expression in ovarian cancer can result in increased cisplatin resistance due to activation of the PI3K/AKT pathway, which is mediated by sponging off the PTEN transcription factor [80, 87, 88, 102, 103]. Furthermore, the microRNAs miR-21, miR-93, miR-130a, and miR-205 can adversely affect the expression of PTEN, which makes OC cells resistant to the chemotherapy drug cisplatin [71, 79, 86, 104]. miR-125b is abundantly expressed in cisplatin-resistant OC cells, and it specifically targets the BAK1 gene, which results in the induction of resistance to cisplatin [82]. When activated, BAK1 increases mitochondrial permeability and stimulates the release of cytochrome C, hence increasing the likelihood of mitochondrial death in the cells that express it [105]. PRKCD is thought to cause apoptosis in cells [106]. Cisplatin resistance in EOC is caused by miR-204, which is a critical component of the IL-6R/STAT3/miR-204 feedback loop [85]. The binding of interleukin-6 to the interleukin-6 receptor activates JAK2, which increases the amount and nuclear translocation of p-STAT3, which promotes the transcription of anti-apoptotic proteins in the cell (BCL-2, survivin, and MCL-1). A second binding site for miR-204 is at the promoter region of TRPM3, where it inhibits the transcription of miR-204, which subsequently increases the expression of its target protein IL-6R, thereby triggering the transcription of anti-apoptotic proteins generated by STAT3 to aid in cisplatin resistance. Increased expression of miR-630 in paclitaxel-resistant ovarian cancer cells results in inhibition of apoptosis by straightly inhibiting the apoptosis-inducing factor (APAF-1) transcription factor [74, 107]. APAF-1, which acts as an activator of mitochondrial apoptosis, causes apoptosis in OC cells. The overexpression of miR-223 and miR-205 in an ovarian tumor xenograft mice model led to a reduction in tumor growth and the expression of the PTEN gene [86, 88].

Similarly, excessive expression of miR-204 boosted cisplatin resistance, whereas decreased expression of miR-630 increased paclitaxel sensitivity in an ovarian tumor xenograft mice model, both beneficial [74, 85]. microRNAs 204 and 630 both influence the malignant phenotype of tumor cells. According to the researchers, ovarian cancer cells are prevented from committing suicide by miRNA mainly by suppressing the death receptor pathway, inhibiting the mitochondrial apoptosis pathway, and stimulating the PI3K/AKT pathway. Although these three signaling pathways have been widely researched, the role of microRNAs in these pathways appears to be a relatively untapped field of research. Investigations on this hallmark will better understand medication resistance in ovarian cancer caused by apoptosis inhibition in the tumor [52].

Concerning ovarian cancer, miRNAs’ differential expression can be a two-edged sword. When it comes to chemotherapy in the mentioned malignancy, paclitaxel and platinum are two types of medications that have been thoroughly investigated to determine whether miRNAs impact both the sensitivity and resistance to chemotherapy. miRNAs can modify the sensitivity of ovarian cancer cells to chemotherapy when they are upregulated or downregulated, respectively (Table 2).

**Table 2:** The use of microRNAs to combat OC drug resistance

MicroRNA Nomenclature	function	target	Resistance against	Reference
miR-30a-5p	Inhibition of migration and invasion	SKP2, BCL9, and NOTCH1	Cisplatin	[108]
miR-34a	Inhibition of proliferation	HDAC1	Cisplatin	[109]
miR-34a-5p	Inhibition of proliferation and G1-phase cell cycle	PD-L1	Cisplatin	[110]
miR-98-5p	Promotion of drug resistance	miR-98-5p/Dicer1/miR-152	cisplatin	[111]
miR-136	Inhibition of proliferation	Notch3	Paclitaxel	[112]
miR-142-5p	Inhibition of drug resistance	XIAP, BIRC3, BCL2, BCL2L2, and MCL1	Cisplatin	[113]
miR-206	Inhibition of proliferation and metastasis	c-Met/AKT/mTOR Signaling Pathway		[70]
miR-338-3p	Inhibition of proliferation, motility, and EMT	WNT2B	Cisplatin	[114]
miR-383-5p	Tumor suppressor	TRIM27	Paclitaxel	[115]
miR-503-5p	Inhibition of tumor angiogenesis and growth	CD97-Mediated JAK2/STAT3 Pathway	Paclitaxel	[116]
miR-509-3p	Enhance drug sensitivity	GOLPH3 and WLS	Platinum	[117]
miR-708	Inhibition of metastasis	IGF2BP1/Akt	Cisplatin	[118]
miR-744-5p	Promotion of cell apoptosis	NFIX and HNRNPC	Carboplatin	[119]
miR-1246	Promotion of tumor growth	Cav1/p-gp/M2-type macrophage axis	Paclitaxel	[120]
miR-1307	It affects cell cycle dynamics	CIC	Paclitaxel	[94]

Neoadjuvant chemotherapy (NACT) for patients with advanced EOC has long been acknowledged as a safe and effective treatment option. There has been no investigation into the molecular pathways that lead to a platinum reaction in NACT environments. In a study of HGSOC patients treated with NACT, researchers discovered that the expression levels of the microRNAs miR181a-5p, miR-199a-3p, miR-199a-5p, and miR let-7G-5p are all self-sufficiently linked with overall survival and PFS (progression-free survival) [121]. Furthermore, the four miRNAs described above are associated with Pt-based resistance and prognosis in patients. The simultaneous expression of miR181a-5p and P-Smad2 in surgical samples could be proficient in approving a weak result and a low likelihood of responding to platinum-based NACT therapy. Through the IGF2BP1/Akt pathway, miR-708 intensifies the sensitivity of cisplatin-resistant cells [118]. In OC cells, miR-34a inhibits proliferation and reduces cisplatin resistance via downregulating HDAC1 expression [109] and targeting the Notch-3 oncogene; miR-136 re-sensitizes OC cells to paclitaxel [112]. When it comes to OC, the expression of miR-383-5p is downregulated, whereas the expression of TRIM 27 is elevated [115]. As a result of decreasing TRIM27 expression, miR-383-5p reduces cell growth and increases chemosensitivity to paclitaxel. In ovarian cancer, oncogenic miR-1246 has been discovered, and its inhibitor has a significant paclitaxel sensitization effect, which is remarkable [120]. Recently, it was shown that miR-503-5p is responsible for inducing metastasis in chemoresistant OC cells [116]. Inhibition of the CD97-mediated JAK2/STAT3 pathway by miR-503-5p prevents the colonization and metastasis of paclitaxel-resistant colon cancer cells. A novel signaling axis known as miR-141/KLF12/Sp1/survivin can increase OC drug resistance, which may be a viable therapeutic target for metastatic OC [122]. Furthermore, miR-200c has been postulated as a possible circulating biomarker in ovarian cancer to predict the prognosis of bevacizumab in combination with standard chemotherapy versus standard chemotherapy alone in patients with advanced cancer [123].

Let-7 g, when utilized as a tumor suppressor, could be used to lower tumor development and cisplatin resistance in epithelial ovarian cancer patients [63]. Snail plays a critical role in epithelial-mesenchymal transition regulation. Remarkably, the tumor suppressor gene let-7 expression was increased in knockout snail cells. Cited data indicate that the Snail/Let-7 axis may be an attractive therapeutic target for high-grade serous ovarian cancer [124]. Compared to conventional tumor suppressors, miR-98-5p, as a member of the let-7 family, has the most inhibitory effect on Dicer1 and is considerably increased in cisplatin-resistant epithelial ovarian cancer cell lines [111]. miR-98-5p contributes to cisplatin resistance via a new miR-98-5p/DICER1/miR-152 pathway. The findings, as mentioned above, may contribute to the development of novel prognostic and predictive models for ovarian cancer and aid in the development of novel miRNA-based treatment methods.

Methods for miRNA detection are constantly being improved. Ongoing research strives to develop a plan of administering drugs that minimizes their local buildup, systemic toxicity, and adverse effects. A novel method of targeted therapy in the treatment of OC is porous anti-miRNA nanoparticles [125]. The exact mechanism through which each microRNA could be used to overcome drug resistance is unknown. In brief, the discovery of microRNAs and their use in the pathophysiology of ovarian cancer open the door to an infinite number of opportunities to translate miRNA scientific research into therapeutic administration.

## Conclusion and future perspective

Ovarian cancer patients are treated chiefly with systemic chemotherapy, the gold standard treatment method. Unfortunately, drug resistance is unavoidable in cancer treatment administered over an extended time. According to preliminary findings, interventions targeting dysregulated non-coding RNAs (ncRNAs) have shown promise in overcoming the medication resistance of ovarian cancer. To achieve ncRNA-targeted therapy, several approaches have been proposed, including the upregulation of ncRNAs through the use of mimics, the exogenous expression or downregulation of ncRNAs through the use of small interfering RNAs (siRNAs) or short hairpin RNAs (shRNAs), and the inhibition of ncRNA function through the use of antisense oligonucleotides. Using these technologies, we will be able to modulate the expression of non-coding RNAs and restore the drug sensitivity of treatment-resistant ovarian cancer cells. Because of this, the combination of ncRNA-targeted therapy with chemotherapy may prove to be a promising approach for the treatment of ovarian cancer shortly. Although it is possible to correctly and successfully apply these non-coding RNA modulators to the human body, this is a huge hurdle. It has been observed that the inclusion of specific oligonucleotides into nanoparticles can boost their delivery efficiency, allowing for the achievement of the best possible therapeutic effect on tumors in the process [126]. As a bonus, GalNAc-siRNA conjugates have been demonstrated to act as siRNAs against mRNAs in cells accurately; as a result, this technology may be studied to treat ovarian cancer medication resistance [127]. However, it is necessary to conduct safety and feasibility studies on such technology before applying it to patients in clinical settings. Increasing the number of relevant clinical trials is essential to grasp the true therapeutic potential of the non-coding RNAs fully and, most importantly, microRNAs to create novel and successful treatment methods for ovarian cancer.

## Data Availability

Not applicable.

## Abbreviations

OC: Ovarian cancer
EMT: Epithelial–mesenchymal transition
CSCs: Cancer stem cells
HGSOC: High-grade serous ovarian cancer
OCCC: Ovarian clear cell carcinoma
LMP: Low malignant potential
BRCA: Breast Cancer gene
PFS: Progression-free survival
ORR: Objective response rate
HR: Hazard ratio
DDR: DNA damage repair
ATP7A: Copper-transporting ATPase 1
CTR1: Copper transporter 1
HMGA2: High Mobility Group AT-Hook 2
LIN28B: Lin-28 Homolog B
C-Myc: C-myelocytomatosis oncogene
IGF2BP1: Insulin Like Growth Factor 2 MRNA Binding Protein 1
ncRNA: Noncoding RNA
circRNA: Small nuclear RNAs
PTEN: Phosphatase and tensin homolog;
PDCD4: Programmed Cell Death 4
BAK1: BCL2 Antagonist/Killer 1
MCL1: Myeloid-cell leukemia 1
MST1: Macrophage Stimulating 1
SAV1: Salvador Family WW Domain Containing Protein 1
YAP: Yes-associated protein
IL: Interleukin
STAT3: Signal transducer and activator of transcription 3
ABCG2: ATP Binding Cassette Subfamily G Member 2
SFRP1: Secreted Frizzled Related Protein 1
HIPK2: Homeodomain Interacting Protein Kinase 2
MDR: Multidrug resistance
BAG3: BAG Cochaperone 3
BCL: B-cell lymphoma/leukemia
APAF1: Apoptotic protease activating factor 1
CIC/DUX4: Capicua–double homeobox 4
ETV: ETS translocation variant 4
SLC27A2: Solute carrier family 22 (organic cation transporter), member 2
ABCC1: ATP Binding Cassette Subfamily C Member 1
ABC: ATP-binding cassette
LEF/TCF: Lymphoid enhancer factor/T-cell factor
GSK3h: Glycogen synthase kinase 3 h
LAST1/2: Large tumor suppressor homolog 1/2
PRKCD: Protein kinase C delta type
TRPM3: Transient Receptor Potential Melastatin 3
SKP: S-phase kinase-associated protein 2
NOTCH1: Notch homolog 1, translocation-associated (Drosophila)
HIDAC1: Histone deacetylase 1
PD-L1: Programmed death-ligand 1
XIAP: X-linked inhibitor of apoptosis protein
BIRC: Baculoviral IAP Repeat Containing 3
BCL2L2: BCL2 Like 2
mTOR: Mammalian target of rapamycin
WNT2B: Wnt family member 2B
TRIM27: Tripartite motif-containing 27
JAK: Janus kinase
WLS: Wntless
GOLPH3: Golgi Phosphoprotein 3
HNRNPC: Heterogeneous Nuclear Ribonucleoprotein C
NFIX: Nuclear Factor I X; CAV1: Caveolin 1
P-gp: P-glycoprotein 1
NACT: Neoadjuvant chemotherapy
EOC: Epithelial ovarian cancer
HGSOC: High-grade serous ovarian carcinoma
KLF12: Kruppel Like Factor 12
Sp1: Specificity protein
siRNA: Small interfering RNA
shRNA: Short hairpin RNA
GalNAc: N-Acetylgalactosamine
